# The Development and Treatment of Lymphatic Dysfunction in Cancer Patients and Survivors

**DOI:** 10.3390/cancers12082280

**Published:** 2020-08-14

**Authors:** Melissa B. Aldrich, John C. Rasmussen, Caroline E. Fife, Simona F. Shaitelman, Eva M. Sevick-Muraca

**Affiliations:** 1 Center for Molecular Imaging, The Brown Foundation Institute of Molecular Medicine, The University of Texas Health Science Center, Houston, TX 77030, USA; Melissa.B.Aldrich@uth.tmc.edu (M.B.A.); John.C.Rasmussen@uth.tmc.edu (J.C.R.); 2 Department of Geriatrics, Baylor College of Medicine, Houston, TX 77030, USA; cfife@intellicure.com; 3 The Wound Care Clinic, CHI St. Luke’s Health, The Woodlands Hospital, The Woodlands, TX 77381, USA; 4 Department of Radiation Oncology, The University of Texas MD Anderson Cancer Center, Houston, TX 77030, USA; SFShaitelman@mdanderson.org

**Keywords:** lymphedema, lymphatic imaging, near-infrared fluorescence imaging, indocyanine green, breast cancer-related lymphedema, head and neck cancer

## Abstract

Breast-cancer-acquired lymphedema is routinely diagnosed from the appearance of irreversible swelling that occurs as a result of lymphatic dysfunction. Yet in head and neck cancer survivors, lymphatic dysfunction may not always result in clinically overt swelling, but instead contribute to debilitating functional outcomes. In this review, we describe how cancer metastasis, lymph node dissection, and radiation therapy alter lymphatic function, as visualized by near-infrared fluorescence lymphatic imaging. Using custom gallium arsenide (GaAs)-intensified systems capable of detecting trace amounts of indocyanine green administered repeatedly as lymphatic contrast for longitudinal clinical imaging, we show that lymphatic dysfunction occurs with cancer progression and treatment and is an early, sub-clinical indicator of cancer-acquired lymphedema. We show that early treatment of lymphedema can restore lymphatic function in breast cancer and head and neck cancer patients and survivors. The compilation of these studies provides insights to the critical role that the lymphatics and the immune system play in the etiology of lymphedema and associated co-morbidities.

## 1. Introduction

**Figure 1:**
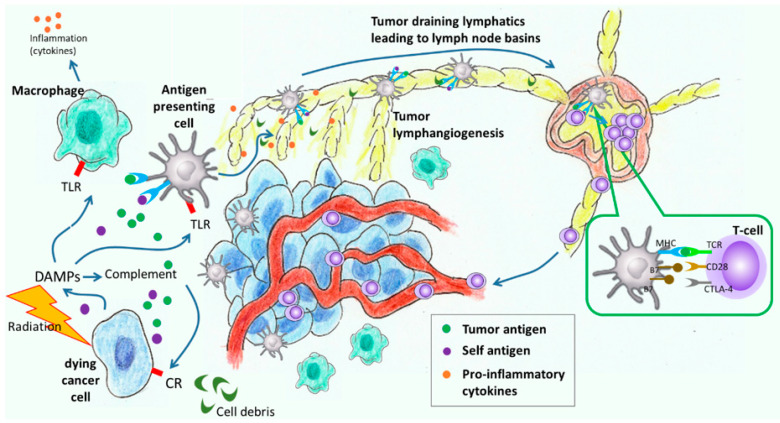
Innate and adaptive immune responses to cancer radiation treatment in which innate immune response results in cell debris, damage-associated molecular patterns (DAMPs), and tumor antigens that activate (i) macrophages to create a pro-inflammatory environment and (ii) dendritic cells (antigen presenting cells) for antigen uptake. Cell debris, cytokines, and dendritic cells are taken up by initial lymphatics and the dendritic cells mature as they travel through the lymphatics to tumor draining lymph nodes (LNs) to educate and activate T cells for tumor immunity. T cells proliferate in LNs, leave through efferent lymphatics and enter blood circulatory system where they home to the tumor microenvironment.

The immune system is well known to play significant roles in cancer progression and treatment. Many cancers metastasize through the lymphatics to regional draining lymph nodes (LNs), where immune priming against cancer can be interrupted and/or immune tolerance to cancer can be established–both resulting in immunological hijacking that facilitates metastatic spread [1]. Radiation treatment (RT) causes tumor cell death and release of damage-associated molecular patterns (DAMPs), which initiates innate immune responses that require functional lymphatics for regionally resolution [2]. Ironically, cancer staging frequently involves LN dissection prior to RT, potentially removing the very sites needed for resolution of innate immune responses (Figure 1). The pro-inflammatory conditions created by RT can also adversely limit lymphatic function, further exacerbating lymphatic insufficiencies caused by LN dissections [3,4]. Metastasizing cancer cells can “block” lymphatic flow, causing lymphatic remodeling and edema [5,6,7]. Consequently, the processes involved in metastatic spread and RT themselves cause adverse lymphatic remodeling that impacts regional lymphatic watersheds.

**Figure 2:**
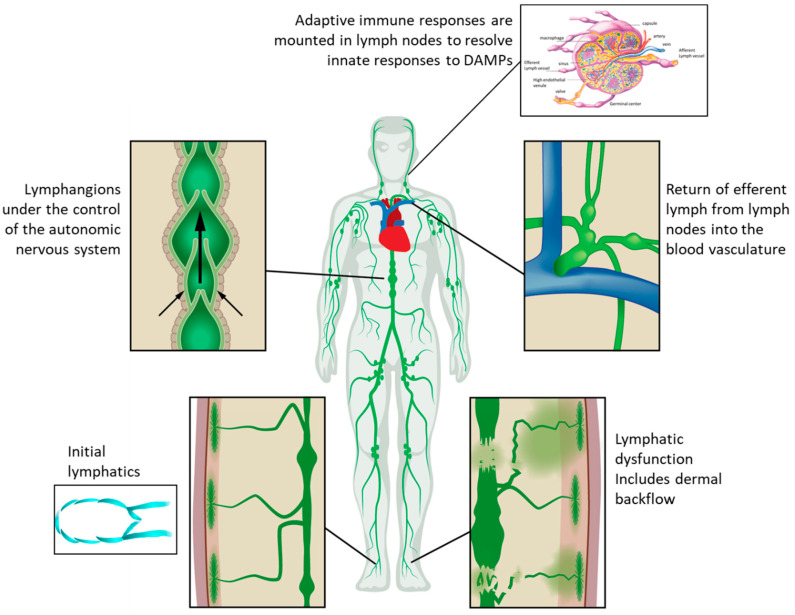
The open, unidirectional lymphatic system wherein entry into the lymphatics occurs at the initial lymphatics that lie beneath the epidermis and surround all internal organs. Once in the initial lymphatics, lymph is transited through collecting and conducting vessels through lymphangions and LNs before return to the subclavian vein (adapted from [10], O’Donnell, T.F., et al. 2017).

Within the past decade, paradigm-changing discoveries have dramatically revised how we view the role of lymphatics in health and an array of chronic conditions, including cancer-acquired lymphedema (LE). In 2010, Levick and Michel showed that capillary filtrate carrying oxygen and nutrients to interstitial tissues is returned along with cellular waste products primarily through the lymphatics, not through the venules as predicted by Starling’s principle of 1896 [8]. The ramifications are that up to eight liters per day of capillary filtrate/interstitial fluid are picked up by the initial lymphatics that line all organs and are returned by the unidirectional lymphatics to the blood circulatory system at the level of the subclavian vein (Figure 2) [9]. Thus, disruption of lymphatics will cause edema and regional accumulation of cellular waste products that could promote inflammatory immune responses.

To maintain immunity and fluid homeostasis, recovery of lymphatic function during and after cancer treatment is essential to health. In this review, we focus on changes in lymphatics due to cancer progression and treatments as visualized by near-infrared fluorescence lymphatic imaging (NIRF-LI). Using this technology, lymphatic responses in cancer patients and in preclinical animal models can be longitudinally monitored to understand the functional lymphatic changes that precede clinical symptoms of LE. Finally, by visualizing changes in lymphatic function with LE treatment, more effective strategies to treat, manage, and possibly cure cancer-acquired LE could result. Herein, we first introduce the basic fundamentals of indocyanine green (ICG) lymphography or the custom near-infrared fluorescence lymphatic imaging (NIRF-LI) used in our studies. We show how trace administration of ICG can provide longitudinal information about lymphatic dysfunction. We next describe the lymphatic anatomy in upper and lower extremities, as well as in the head and neck region, before showing NIRF-LI images and videos that reveal changes in lymphatics with cancer progression, cancer treatment, and onset of LE. We also present results showing how NIRF-LI can direct treatment of LE and describe unexplored research and advances that are needed to eliminate the burden of cancer-acquired LE and its co-morbidities. 

### 1.1. Near-Infrared Fluorescence Lymphatic Imaging (NIRF-LI)

**Figure 3:**
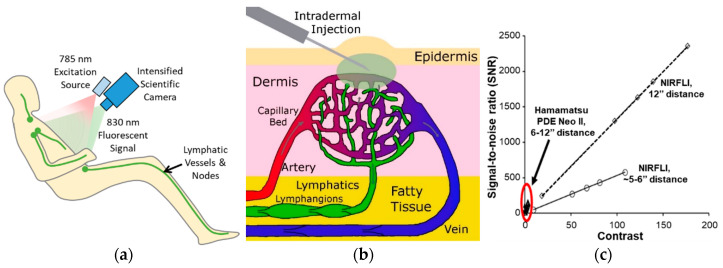
(**a**) Schematic of the imaging device and the typical lymphatic drainage patterns in humans. (**b**) Schematic of the dermal lymphatic vasculature and its relationship to the blood vasculature as well as the indirect delivery of contrast agent to the lymphatic plexus via intradermal injection. (**c**) Plot of the signal-to-noise ratio and contrast of the custom near-infrared fluorescence lymphatic imaging (NIRF-LI) device and a Hamamatsu PDE Neo II commercially available device.

Routine lymphatic imaging in peripheral watersheds is conducted with the off-label intradermal administration of 0.1 cc of 0.25 mg/ml indocyanine green (ICG), illumination of tissues with dim, 785 nm near-infrared light, and collection of the near-infrared (NIR) fluorescence (830 nm) emanating from tissues (Figure 3a). Taken up by the initial lymphatics beneath the epidermis (Figure 3b), ICG injections can be made anywhere on the body, enabling visualization of functional lymphatic drainage to major LNs as deep as 3–4 centimeters beneath the tissue surface [11]. Because conventional silicon (Si)-based charge-coupled device (CCD) and complementary metal oxide semiconductor (CMOS) detectors are relatively insensitive to NIR light, our team has built and deployed gallium arsenide (GaAs)-intensified CCD and CMOS systems for rapid, millisecond-based imaging with NIRF-LI that allows dynamic imaging of lymph movement within a watershed to draining LNs. When comparing device performance to other Si-based devices through traceable standards, we have shown NIRF-LI outperforms Si-based systems with superior contrast and signal-to-noise ratio that allows for detection [12]. This comparison includes recent devices used in lymphovascular surgeries (Figure 3c). In addition, while recent advances in indium (In)GaAs array detectors can detect the short-wave infrared fluorescence (1064 nm) that can result from 785 nm excitation of ICG, we have shown that GaAs-intensified Si-based detection of NIRF light (~830 nm) remains the most sensitive [13]. There are similar approaches, termed ICG lymphography, that use different device designs using higher doses of ICG for imaging lymphatic anatomy. Herein, we review our experiences with breast and head and neck cancer patients using NIRF-LI systems.

**Figure 4:**
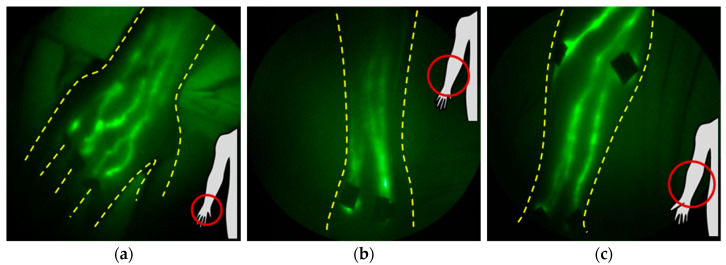

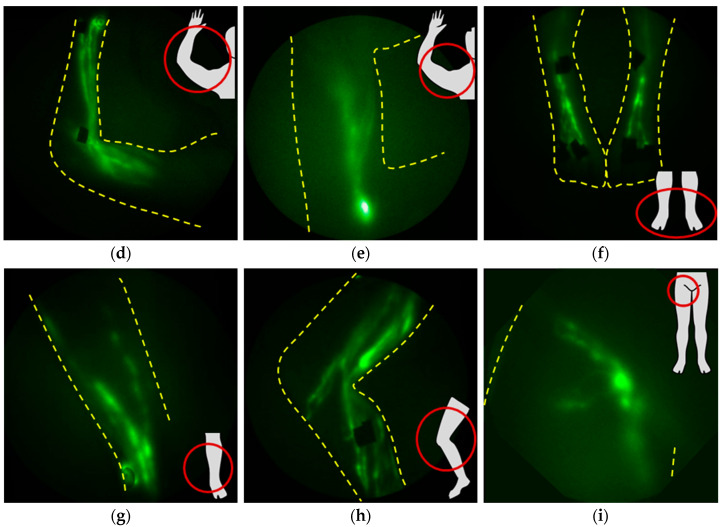
Typical NIRF-LI images of normal lymphatics in the upper and lower extremities including the (**a**) dorsum of the hand, (**b**) dorsal and (**c**) volar forearms, (**d**) medial arm, (**e**) axillary nodes, (**f**) dorsum of the feet, (**g**) medial ankle, (**h**) medial calf and knee, and (**i**) inguinal basin. Injection sites are covered with round bandages and/or black vinyl tape. The brightness and contrast of the images have been adjusted to facilitate the visualization of the 16-bit imaging depth.

Figure 4 (plus associated video) depicts NIRF-LI of normal lymphatic function and anatomy in the upper and lower extremities. In contrast to the radioactive contrast used in conventional lymphoscintigraphy, ICG does not need to be injected into the interdigital web spaces, as intradermal injections in the dorsal aspects of hands and feet are effective for visualizing major lymphatic watersheds. Under normal healthy conditions, ICG does not radially spread interstitially directly from the injection site, but rather ICG-laden lymph enters into conducting vessels and is pumped sequentially across a series of lymphangions, which are segmented lymphatic vessel structures bounded by valves and surrounded by smooth muscle. The frequency of lymphangion pumping in healthy control arms and legs is generally between 0.8 ± 0.4 pulses/minute and 0.9 ± 0.7 pulses/minute, respectively [14]. The frequency of contractile activity does not appear to be related to heartbeat or respiration, but can be enhanced by motion or, anecdotally, through deep breathing exercises. The mechanisms that mediate the contractile activity of lymphangions are termed extrinsic/passive and intrinsic/active and are under ongoing investigation. It is suspected that the autonomic nervous system may be responsible for lymphatic pumping [15].

ICG lymphangiography provides similar imaging to NIRF-LI, most often with greater ICG dosage and reduced performance as shown in Figure 3c and elsewhere [12,16]. The low dose of ICG allows NIRF-LI visualization of the contractile activity that can be “swamped” by fluorescent signals with higher doses. Thus, the sensitivity of NIRF-LI affords the ability to image dynamic lymphatic function with timescales on the order of 10s–100s of milliseconds to provide quantitation of contractile activity and visualization of lymphatic “reflux” not possible in conventional radiology procedures. Additionally, the intradermal depot of ICG provides a source of contrast for several hours. Conventional imaging of the lymphatics with lymphoscintigraphy (conducted with radionuclide administration) or lymphangiography (conducted with administration of Gadolinium or iodinated contrast for magnetic resonance (MR) or computed tomography (CT) detection) provide static imaging and cannot be easily repeated in longitudinal studies. However, neither ICG lymphangiography nor NIRF-LI are capable of imaging the thoracic duct or deep truncal lymphatics, as can be probed with MR or CT lymphangiography in syndromic conditions. Readers are referred to reviews of medical imaging of the lymphatics for more details on these techniques [17,18,19,20]. As a non-radioactive, non-ionizing imaging technique, NIRF-LI lends itself well to “point-of-care” use for repeated, longitudinal imaging, as shown below. Photoacoustic imaging is another emerging optical technique that likewise uses ICG lymphatic contrast to generate superficial 3D images of lymphatic vessels, but can be limited by the need to perform time-consuming, pulsed laser scanning that is unable to provide dynamic functional imaging [21]. Finally, while ICG is a dim fluorophore with a small Stoke’s shift and fluorescent yield that can limit detection, it clears through the hepatobiliary system within two minutes after reaching the blood vasculature. As a result, the exceptional safety record of ICG in the clinic makes it unlikely that it will be replaced by better-performing fluorescent imaging agents with greater Stoke’s shifts and higher fluorescent yields [22]. When ICG is used as an investigational contrast agent administered off-label, either intradermally or intramucosally for lymphatic imaging, a common exclusion criterion is an allergy to iodine, typically indicated by an existing shellfish allergy. Inclusion criteria include appropriate cancer or lymphedema diagnosis, appropriate cancer treatment, absence of interfering tattoos, ambulation, and ability to lie supine for imaging. Exclusion criteria include weight over 300 pounds and pregnancy or trying to become pregnant.

### 1.2. Anatomy and Function of Upper and Lower Extremity Lymphatics

The medial lymphatics that drain the breast empty into the axillary, infraclavicular, and intermammary LNs. The axillary LNs also receive lymph from the ipsilateral arm, starting from the distal, dense network of lymphatics in the hand (Figure 4a). From there, lymph is actively pumped to the dorsal aspect of hand through the dorsal (Figure 4b) and volar (Figure 4c) lymphatics in the wrist and to the medial lymphatic bundles that wrap from the posterior to anterior upper arm (Figure 4d) into the axillary lymph nodes (Figure 4e) and infraclavicular lymph nodes [23,24]. Video S1 shows lymphatic pumping in a normal healthy arm. In the lower extremities, the dense network in the foot drains to the dorsal aspect of the foot (Figure 4f) and lymph is propelled through lymphangions into the superficial lateral bundles (Figure 4g,h) accompanying the saphenous veins, as well as the posterior tibial and peroneal lymphatics, before draining into the inguinal LNs (Figure 4i). From the inguinal LNs, lymph is transited to the external and internal iliac, preaortic, and aortic LNs that may be removed as part of treatment in melanoma, genitourinary, and gynecological cancers. Intradermal injections on the dorsal hand or foot result in ICG drainage that enables imaging of axillary or inguinal LNs.

### 1.3. Anatomy and Function of Cranial/Cervical Lymphatics

**Figure 5:**
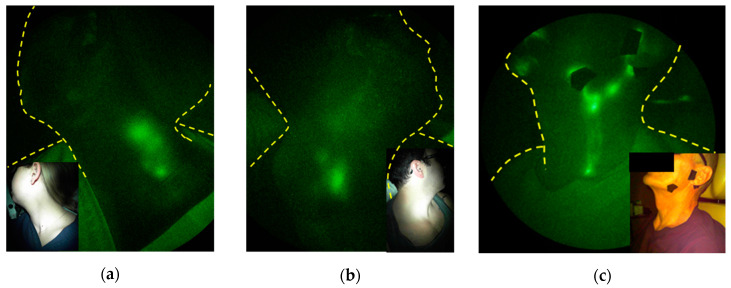
Typical NIRF-LI images of the lympho-jugular lymphatics draining to the (**a**) cervical and (**b**) supraclavicular lymph nodes following the injection of contrast agent into the palatine tonsils and (**c**) the external lymphatics draining to the supraclavicular lymph nodes following facial intradermal injections (adapted from [29], Rasmussen, JC, et al. 2018). Injection sites are covered with round bandages and/or black vinyl tape. The brightness and contrast of the images have been adjusted to facilitate the visualization of the 16-bit imaging depth.

The head and neck region contains one third of the body’s LNs and extensive lymphatic networks that serve as the lymphatic watershed draining the cranium. In adults, the nearly 600 cc of cerebrospinal fluid (CSF) that is produced each day is thought to clear through this network. While the venous arachnoid granulations in the subarachnoid space have been traditionally credited for its clearance, the low transmural pressure suggests little, if any, venous reabsorption occurs [25]. Instead, intrathecal injections of Gd-contrast in humans and of ICG in animals show that cervical lymphatics are the major conduits of CSF outflow [26,27]. In humans, the possible neurological consequences of Gd deposits in the brain limit its use for assessing CSF outflow [28]. While intrathecal ICG administration has not been cleared by the FDA, we have conducted off-label administration of ICG into the mucosal lymphatics of the palatine tonsils under FDA clearance [27,29]. This administration enables imaging of the ipsilateral lympho-jugular lymphatic chain that drains into the cervical (Figure 5a) and supraclavicular LNs (Figure 5b) when subjects are sitting upright. The mucosal lymphatics that line the throat are impacted in head and neck cancer treatments and are commonly referred to as “internal lymphatics,” as they drain near the internal jugular vein [30]. Their dysfunction after cancer treatment may be responsible for swelling of the throat and base of tongue as well as for impairing speaking, breathing, and swallowing in head and neck cancer survivors. Impairment of CSF outflow due to congestion of downstream LN basins could also be responsible for reduced clearance of waste products from the brain and is a topic of active investigation. Indeed, impaired CSF outflow into the lymphatics may be responsible for the accumulation of cytokines and extracellular proteins that characterize aging, neuroinflammation, and neurodegenerative diseases [31,32,33,34,35], although there has been no studies to discern whether survivors with head and neck LE are at increased risks for neurodegenerative diseases such as Alzheimer’s Disease.

In contrast to ICG intramucosally injected in the oral cavity, ICG intradermally administered on the face, generally fore and aft of the ear, chin, cheek, lateral forehead, and along the jawline, drains through the superficial or “external lymphatics” (Figure 5c). These external lymphatics include the submental nodes that drain the skin of the chin, lower lip, floor of the mouth, and anterior portion of the tongue; the supra- and sub-mandibular nodes that drain the skin and mucosa of the cheeks, upper and lower lips, and floor of the mouth; the posterior nodes that drain lateral and posterior neck and the nasopharynx, oropharynx, and thyroid; and the superficial and deep cervical nodes that receive drainage from above [30]. These lymphatics may become compromised with surgical incision and RT, resulting in swelling of lips, under the chin, along the jawline, cheeks, and eyelids, commonly recognized as head and neck LE. It is noteworthy that both external and internal lymphatic pathways reach the supraclavicular LNs. Head and neck cancer (HNC) LE is estimated to impact 75% of all HNC patients and is underdiagnosed due to the lack of objective measures [36].

### 1.4. NIRF-LI Taxonomy of Lymphatic Dysfunction 

**Figure 6:**
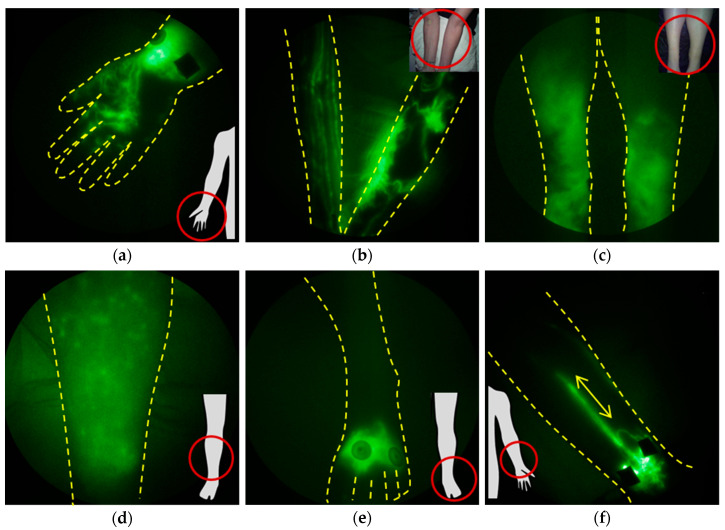
Typical NIRF-LI images of lymphatic dysfunction. The retrograde movement of contrast agent into the dermal lymphatics, known as dermal backflow, is the most common sign of dysfunction and is shown here (**a**) around the injections in the wrist and into the hand and fingers. (**b**) Dermal backflow and tortuous lymphatic vessels present in the affected arm of a subject but not the contralateral asymptomatic arm. (**c**) Extensive dermal backflow in both lower legs of a subject. (**d**) Extravascular dye is sometimes observed in the interstitial space in some subjects. (**e**) Radial movement of dye around the injection sites is also observed in some subjects and is often one of the first anatomical signs of dysfunction. (**f**) Lymphatic reflux is observed, indicating that lymphatic valve dysfunction may occur, in some subjects (adapted from [14], Rasmussen JC, et al., 2010). Injection sites are covered with round bandages and/or black vinyl tape. The brightness and contrast of the images have been adjusted to facilitate the visualization of the 16-bit imaging depth.

Lymphatic dysfunction is a harbinger of cancer-acquired LE, but it is also a harbinger of several other common chronic conditions (as discussed in Section 4). One of the most discernable and earliest-appearing features of lymphatic insufficiency observed with NIRF-LI is dermal backflow (Figure 6a,b). Dermal backflow occurs when lymph flows from conducting vessels backward toward the collecting and initial lymphatics, indicative of upstream obstruction or insufficient lymphangion pumping pressures to maintain distal-to-proximal flow toward the trunk of the body. In our studies, regions of dermal backflow connected by dilated and/or tortuous, semi-functional lymphatic vessels (Figure 6b) provide an early, subclinical sign of lymphatic dysfunction that precedes swelling. Dilated vessels may fill with ICG, but active contractile function is often missing or reduced. With onset of edema, the degree of coverage of dermal backflow increases (Figure 6c), and dermal backflow can result in extravascular leakage of lymph into the epidermis, as shown in the distinctive pattern in Figure 6d. In cases in which there is impaired uptake into the initial lymphatics, NIRF-LI shows radial spread of ICG accumulation at the site of intradermal injection (Figure 6e). Video S2 exhibits dysfunctional lymph flow as shown with NIRF-LI in LE-affected feet. Retrograde flow occurs when lymph flows proximal-to-distal, as shown in the hand in Figure 6a; when retrograde flow occurs intermittently with distal flow within the collecting vessels, the phenotype is termed “lymphatic reflux” (Figure 6f). In all of these stages, lymph is not moved efficiently in a proximal direction toward the trunk of the body for return to blood vasculature at the subclavian vein. Edema, adipose deposition, and ultimately fibrosis results from lymphatic insufficiency, along with the inability to resolve innate or mount adaptive immune responses within the respective lymphatic watershed. These NIRF-LI features of dysfunction are shared not only in persons with cancer-related with LE and congenital LE [14,37,38,39,40,41,42], but with other chronic immune conditions that involve lymphatic dysfunction. ICG lymphography has provided “still” images similar to those seen in images and videos with NIRF-LI, and other groups have adopted classification and staging systems for LE [43,44,45,46,47,48,49,50].

## 2. Lymphatic Responses to Cancer Progression and Cancer Treatment

### 2.1. The Effects of Metastasis and Cancer Progression on Lymphatic Function

While much attention is given to hematological transit of cancer cells, and most chemotherapy is delivered intravenously, metastatic spread is most often initiated through tumor-draining lymphatics to regional draining LNs before draining into and disseminating through the blood circulation. Expanded peritumoral lymphangiogenesis is frequently observed as part of cancer progression and correlates with increased LN metastasis and poorer survival [51,52], further evidencing that the lymphatics provide a highway for system dissemination. However, before cancer cells metastasize through these peritumoral lymphatics, tumor antigens (tAgs) and mature, activated dendritic cells presenting tAgs migrate through the peritumoral lymphatics to regional LNs to set up anti-tumor immune responses (Figure 1). Indeed, lymphangiogenesis and drainage to regional LNs may improve anti-tumor immune responses, as recently demonstrated by enhanced melanoma and breast cancer growth rates in preclinical models in which regional draining lymphatics were ablated [53].

On the other hand, pro-lymphangiogenic factor VEGF-C secreted by tumor cells and proinflammatory cytokines that are expressed by activated immune cells in the tumor microenvironment act to dilate lymphatic vessels, impair lymphatic pumping [54,55], and can potentially limit anti-tumor immune responses. Immunosuppression can occur from co-inhibitory checkpoint signaling in tumor-draining LNs that prevents the maturation and proliferation of T cells against tAgs. Even if T cells are successfully activated against tAgs, proliferate, and leave LNs for systemic dissemination, upon their exit through efferent lymphatic vessels, they can be “tolerized” by the binding of programmed death 1 (PD-1) receptor upregulated on activated T cells, with its ligand (PD-L1) expressed on lymphatic endothelial cells (LECs) [56,57,58]. In manners that are not yet completely characterized, LECs can variably express signaling machinery for Ag presentation and co-inhibitory signaling to “tolerize” cells [59,60,61,62]. As a consequence, the lymphatics, typically a site for initiating anti-tumor immune responses, can also be a site for initiating immune tolerance to cancer. Once tolerance or immunosuppression in regional lymphatics is established, chemotaxis-driven migration of cancer cells into the lymphatics occurs [63], and in-transit lymph vessel or LN metastases can obstruct downstream lymphatics. Consequently, the lymphatics may have a profound effect upon cancer progression and/or arrest of metastasis and may be a target of emerging immunotherapies. Of note, a particularly treatment-resistant form of breast cancer, inflammatory breast cancer, is characterized by direct invasion of tumor emboli that obstruct draining lymphatics [64,65].

**Figure 7:**
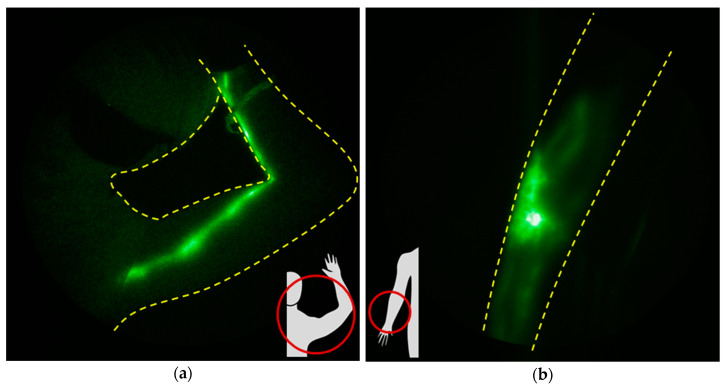
NIRF-LI images of (**a**) dilated lymphatics and (**b**) dermal backflow in the ipsilateral arms of two advanced breast cancer patients who had undergone neo-adjuvant chemotherapy prior to imaging but no surgical or radiological intervention. Injection sites are covered with round bandages and/or black vinyl tape. The brightness and contrast of the images have been adjusted to facilitate the visualization of the 16-bit imaging depth.

NIRF-LI may detect the influence of cancer progression on lymphatics. For example, in advanced breast cancer patients undergoing neoadjuvant chemotherapy, lymphatic vessels are dilated, and regions of dermal backflow in lymphatics draining toward the affected nodal basin may be present, as shown in Figure 7. Such lymphatic dysfunction can occur prior to treatments of surgical dissection and/or RT, suggesting that cancer progression, particularly LN metastasis, contributes to lymphatic dysfunction in cancer patients prior to surgery and RT. While there is evidence that chemotherapy may potentially contribute to the development of cancer-acquired LE [66,67,68,69], further longitudinal studies are needed to understand all the contributing factors that can cause lymphatic dysfunction. Nonetheless, recent results in Figure 7 confirm the results of other studies showing subclinical edema measured with bioimpedance of water content in the arms in breast cancer patients prior to first-line treatment [70,71].

Genetic predisposition for breast-cancer-related LE (BCRL) could also be a contributing factor for the condition. Investigators hypothesize that cancer treatment serves as a “second-hit” in patients with germline mutations associated with pathways associated with lymphangiogenesis [72,73,74]. Using NIRF-LI, we have phenotyped families with rare, non-syndromic congenital LE [38,39,41], and have found occult lymphatic dysfunction in family members who are carriers of rare mutations but are not symptomatic for congenital LE. While these family members were not cancer survivors, and our studies were focused upon rare mutations not found in the general population, the hypothesis of a less-rare, genetic predisposition for cancer-acquired LE that could be phenotyped by NIRF-LI is highly probable. However, determining whether genetics increase risk of LE will need large studies and biological confirmation. Alternatively, imaging lymphatic dysfunction prior to treatment could help prognose risk for developing LE.

To date, we have not imaged dermal backflow in the external lymphatics of treatment-naive HNC patients with known or suspected LN metastases, probably because these *external* lymphatics are typically not in the same lymphatic watershed directly draining interior oropharynx, larynx, and oral cancers. However, in preliminary studies of internal lymphatic drainage of treatment-naive HNC patients, few exhibited drainage into the lympho-jugular chain after mucosal administration of ICG [29], as was seen in normal-health subjects (Figure 4 above) [27]. This observation is preliminary and needs more carefully controlled studies to underscore changes in internal lymphatic drainage from the cranium with head and neck cancer progression. Nonetheless, whether there is “mucosal backflow” in the trachea/airway that is akin to “dermal backflow” in the epidermis requires determining by fluorescent endoscopy.

### 2.2. The Role of Lymph Node Dissection and RT on Lymphatic Function

**Figure 8:**
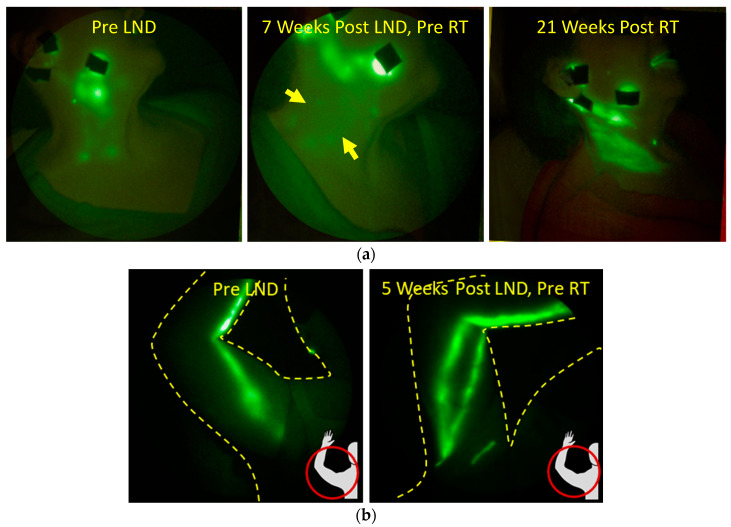
Examples of normal and abnormal lymphatic recovery following LN dissection and radiation treatment (RT). (**a**) NIRF-LI images overlaid on color images showing the case of a head and neck cancer patient who had 19 nodes surgically removed. Approximately seven weeks post-surgery, no dermal backflow was visible and two lymphatic vessels (arrows) with active lymphatic propulsion were faintly visible. Dermal backflow was observed when next imaged approximately 21 weeks after the completion of RT (adapted from [29], Rasmussen, JC, et al., 2017). (**b**) The case of a neo-adjuvant, advanced breast cancer patient before and after LN dissection with no RT. Injection sites are covered with round bandages and/or black vinyl tape. The brightness and contrast of the images have been adjusted to facilitate the visualization of the 16-bit imaging depth.

As assessed in preclinical animal models, surgical disruption of lymphatic vessels or LNs, not in the setting of cancer or RT, yields temporary lymph flow disruption, eventually followed by lymph vessel remodeling and regrowth, with restoration of lymphangion activity [75,76,77,78]. Clinical NIRF-LI results likewise shows normal phenotypes following LN dissection in ~50% of head and neck [29] cancer patients prior to RT (Figure 8a) and in some breast cancer patients (Figure 8b). 

Evidence is accumulating that radiation itself, while efficacious for killing cancer cells and promoting up the immune system for anti-tumor immunity, may damage lymphatics. Treatment of mice with 20 Gy after popliteal lymphadenectomy showed that RT transiently disrupted lymphatic contractile activity, but when radiation was fractionated [4 × 5 Gy] or increased to 40 Gy in one fraction, persistent lymphatic dysfunction occurred, as visualized by NIRF-LI [79]. Strikingly, with increased radiation dose, there was a concomitant increase in lymphatic vessel area in the epidermis, consistent with reports of hyperplasia in the skin of patients after RT [80]. The preclinical imaging results also confirm the hypothesis that radiation causes lymphatic vessel “leakiness,” as demonstrated in rat mesenteric LECs [81] and apoptosis of LECs, with subsequent fibrosis in mouse tails [82]. RT stimulates the innate system through the production of DAMPs, with activation and maturation of macrophages and dendritic cells whose egress through the lymphatic watershed is impaired under conditions of lymphadenectomy. The activated immune cells produce inflammatory cytokines such as tumor necrosis factor-alpha (TNF-alpha) and interleukin-1-beta (IL-1-beta) [3] that are potent inhibitors of lymphangion activity) [4]. Interestingly, the preclinical changes in the lymphatics with radiation after lymphadenectomy are consistent with changes seen with longitudinal imaging in breast and head and neck cancer patients, made possible by repeated NIRF-LI. Whether radiation treatments may be more impactful prior to LN dissection, and whether fractionation schedules can be optimized to reduce collateral lymphatic dysfunction, remain to be investigated.

In an ongoing prospective and longitudinal NIRF-LI surveillance study of breast cancer patients receiving neoadjuvant chemotherapy, axillary lymph node dissection (ALND), followed by RT, we observed dermal backflow pre-ALND and post-ALND, both pre-RT and post-RT (ClinicalTrials.gov, NCT02949726). In almost every case, dermal backflow preceded arm swelling. The variable appearance times for dermal backflow imply that any one (or a combination) of cancer treatment modalities may contribute to lymphatic dysfunction.

In a longitudinal study, Rasmussen et al. [29] imaged head and neck cancer patients before surgery, before RT, and then periodically for up to two years to assess post-therapeutic lymphatic recovery. While none of the patients initially presented with dermal backflow, approximately half developed some dermal backflow near the scar line after surgery but before RT, and nearly all the remaining subjects developed dermal backflow following RT. Because RT was the standard of care for these patients, it is not known whether the pre-RT dermal backflow would have subsequently resolved, as is suggested in the animal studies discussed previously. However, it is noteworthy that the dermal backflow, whether pre- or post-RT onset, was persistent in all patients over months and years after RT therapy.

Whether aberrant lymphatic dysfunction after RT results from the impaired clearance of macrophages and dendritic cells activated by DAMPs, which in turn secrete pro-inflammatory cytokines that impair contractile activity, remains to be investigated. Nonetheless, these results suggest the confounding effect of RT on lymphatic function that may be one of the most significant drivers for lymphatic dysfunction and the onset of cancer-acquired LE.

## 3. Diagnostic Imaging of Dysfunctional Lymphatics for Staging and Treatment of Lymphedema

### 3.1. Diagnosis of Lymphatic Dysfunction and Its Treatment in Patients at Risk for Cancer Acquired Lymphedema

Clinical LE diagnosis can be difficult because classic physical signs (swelling, heaviness) of LE are unreliable—sensitivity and specificity of such clinical signs in predicting lymphoscintigraphy-confirmed LE are 17% and 88%, respectively, with overall accuracy of 47% [83]. Clinical LE diagnosis typically is only made once arm swelling is obvious in breast cancer survivors, or neck swelling manifests for head and neck patients. LE incidence rates vary greatly, depending upon surveillance tools used [84]. Breast cancer LE surveillance tools can deliver varying results—bioimpedance spectroscopy delivered a 36% false negative rate, compared to ICG lymphography [85] in one study of 58 diagnosed LE patients, and a positive predictive value of 61–71%, with a negative predictive value of 67–70%, in a large study of 134 patients diagnosed with LE versus 261 patients without LE [86]. A number of studies report classification systems derived from lymphangiography, lymphoscintigraphy, BIS, or ICG lymphography [87,88,89,90]. Because accurate and early LE diagnosis and treatment result in significantly improved outcomes and quality of life [91,92], our work has focused on early diagnosis of lymphatic dysfunction by NIRF-LI, often prior to onset of clinical symptoms.

As described above, we have identified the imaging phenotype of dermal backflow as one of the earliest signs of lymphatic dysfunction. In the absence of clinically overt swelling, dermal backflow may become an objective, prognostic criterion for development of clinical LE, its sequela, and comorbidities.

**Figure 9:**
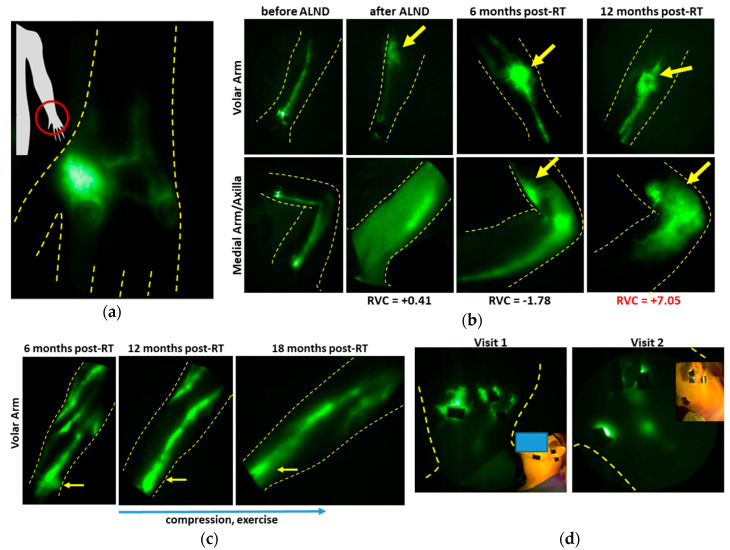
Images of lymphatics in breast (**a**–**c**) and head and neck (**d**) cancer survivors. (**a**) A region of “heaviness” but no swelling manifesting dermal backflow. (**b**) Dermal backflow observed longitudinally before the relative volume change (RVC) is more than 5%, which is the threshold for clinical diagnosis. (**c**) Resolution of dermal backflow after commencement of compression and exercises. (**d**) Resolution of dermal backflow after two weeks of advanced pneumatic compression device (APCD) therapy (adapted from [93], Gutierrez, C., et al., 2019). Injection sites are covered with round bandages and/or black vinyl tape. The brightness and contrast of the images have been adjusted to facilitate the visualization of the 16-bit imaging depth.

For example, in study subjects at risk for unilateral BCRL but without any measurable arm volume change, patient-reported outcome of regional “heaviness” was accompanied by dermal backflow in that region (Figure 9a). In the ongoing longitudinal study of advanced breast cancer patients, NIRF-LI images show dermal backflow that manifests in the absence of arm swelling (Figure 9b), and by the 12 months post-RT surveillance visit, when arm swelling (expressed as relative volume change (RVC)) became clinically evident, lymphatic backflow had been present for over one year. In a contrasting example, Figure 9c chronicles the development and later regression of dermal backflow in a breast cancer patient who independently initiated compression garment wear and exercise at the onset of dermal backflow, and at successive visits six months and 12 months later, the backflow was not evident.

In a case of an HNC patient four weeks after RT, we imaged dermal backflow and poor drainage to cervical LN that, unlike in prior studies of untreated HNC patients, dissipated after two weeks of home treatment with an advanced pneumatic compression device (APCD) (Figure 9d). Indeed, in a pilot study to explore the early intervention by APCD therapy, the surface coverage of dermal backflow decreased over two weeks of treatment, suggesting that impaired lymphatic function was improved or recovered with treatment [93].

Both of these examples in breast and HN cancers suggest that adding diagnostic NIRF-LI to the examination toolbox could prompt more timely initiation of therapeutic strategies at sub-clinical stages of lymphatic dysfunction, and perhaps minimize or even reverse LE development. “Cure” for cancer-acquired LE may require early intervention to reverse lymphatic dysfunction prior to the onset of swelling and accumulation of inflammatory waste products and immune cells. MLD and APCD therapy are generally contraindicated until patients are cancer-free, based on the entirely theoretical concern that they might facilitate cancer spread. The basis for this indication is that metastasis through the lymphatics may be augmented with MLD and APCD therapy. However, as described above, early restoration of drainage to existing or newly formed LNs may prevent LE, and both may potentially enhance the benefits of RT by ensuring anti-tumor immunity.

It is also noteworthy that, in a prior review of NIRF-LI studies of unilateral BCRL patients, contralateral, undiagnosed arms exhibited increasing surface coverage of dermal backflow with stage and duration of diagnosed contralateral LE, even though cancer treatments were confined to the ipsilateral arm [94]. Other studies have found disrupted lymphatic flow on contralateral limbs in unilateral BCRL patients [95,96,97], suggesting unknown mechanisms that may work to progressively deteriorate lymphatics systemically after initial clinical LE diagnosis.

### 3.2. Imaging Lymphatic Response to LE Treatment

While prospective double-blind studies remain to be performed to show that remediation of dermal backflow as an early sign of lymphatic dysfunction can prevent clinical LE, strategic deployment of current treatments for clinical LE may, in the meantime, be enhanced with NIRF-LI diagnostics. Treatments include complete decongestive therapy (CDT), advanced pneumatic compression (with segmented and sequentially inflated chambers), and lymphatic surgeries. Because the intradermal depot of ICG provides a source of contrast for several hours, if not longer, pre- and post-treatment imaging can be performed to assess efficacy.

**Figure 10:**
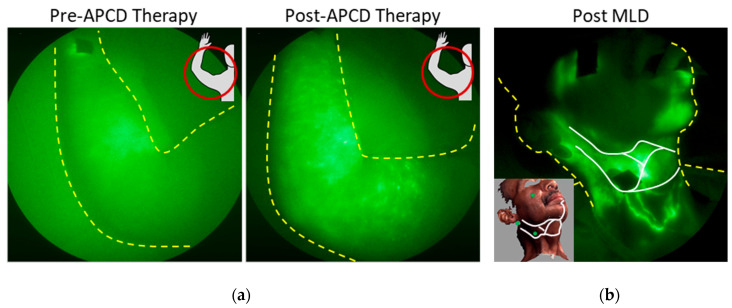
NIRF-LI images illustrating the movement of fluorescent lymph (**a**) through the extravascular space following APCD therapy in a breast-cancer-related lymphedema (BCRL) subject (adapted from [99], Adams, K.E., et al., 2010) and (**b**) across scar tissue following manual lymphatic drainage (MLD) in a head and neck cancer patient (adapted from [100], Maus, E.A., et al., 2012). The white lines in (**b**) represent the location of the scars from multiple surgeries in the neck and the green dots in the inset image show the location of the injection sites. Injection sites are covered with round bandages and/or black vinyl tape. The brightness and contrast of the images have been adjusted to facilitate the visualization of the 16-bit imaging depth.

NIRF-LI has shown that manual lymphatic drainage (MLD), as part of comprehensive CDT, can stimulate lymphatic contractile function by the lymphangions in the affected limbs of patients with diagnosed LE, but is more effective in contralateral “normal” limbs [98]. One may expect that lymphatic contractile function seen through NIRF-LI may be more readily stimulated at early stages of lymphatic dysfunction, than at later stages, consistent with enhanced efficacy of MLD at early stages of diagnosed LE. In BCRL patients without apparent uptake of intradermally administered ICG, APCD therapy moves ICG-laden lymph extravascularly toward draining lymph nodes (Figure 10a) [99]. In an advanced head and neck LE patient, NIRF-LI showed newly formed functional lymphatic vessels across fibrotic surgical scar lines enabling successful re-direction of MLD toward those functional lymphatics (Figure 10b) [100].

When conventional CDT fails, surgical options may improve quality of life for LE patients. Surgeries include the LYMPHA preventive technique, which is performed at the same time as ALND, and connects transected main lymphatic trunks to a lateral branch of the axillary vein distal to a competent valve [101]. Two other physiological surgeries that strive to restore lymphatic vessel networks after breast cancer treatment—lymphovenous bypass (LVB), which avoids drainage to LNs by shunting the lymph directly to the veins, and vascularized LN transfer (VLNT) are frequently performed on patients diagnosed with LE. Patient demand for these microsurgeries has increased steadily over the past few years, driven by the lack of other effective treatments for LE. While these procedures reduce swelling by an average of 25–46%, decrease symptom burden, lessen risk of progression to chronic LE, improve function and cosmesis, and decrease amount of time spent daily on therapy, they do not usually eliminate the need for daily maintenance therapy, such as bandaging [102,103,104,105,106]. Remarkably, though, LVB and VLNT patients experience significantly decreased cellulitis incidence following surgery [105,106,107,108,109,110,111]. Because of the variable reductions in limb swelling and the continued need for daily maintenance therapy, use of the microsurgeries in LE patients remains controversial. In general, like conventional treatments, lymphovascular surgery is most effective when performed in earlier stages of LE, while there is still capacity for recovery of the lymphatic system [106]. Studies using ICG lymphography suggest that subjects with minimal dermal backflow are excellent candidates for lymphovascular surgeries. However, given that dermal backflow may be reversed through conventional treatments as shown in Figure 9, it is unclear that dermal backflow alone is an adequate criterion for early intervention with surgical treatment. Disappearance of dermal backflow, recruitment of new lymphatic vessels, and restoration of drainage to existing or new LNs could be diagnostic hallmarks of surgical success and provide evidence of benefit. Studies using NIRF-LI to objectively measure these phenotypes as lymphosurgical outcomes are underway.

## 4. Conditions and Comorbidities Associated Lymphatic Dysfunction and Lymphedema

Other common conditions result in NIRF-LI features of dermal backflow and reflux as seen in early stages of cancer-acquired LE, and in advanced stages, result in LE diagnoses.

### 4.1. Chronic Venous Disease (CVD)

Chronic venous disease encompasses a wide range of lower extremity symptoms ranging from unsightly superficial telangiectases to chronic venous insufficiency that, if left untreated, can progress to venous ulceration. We have shown that patients with venous leg ulcers have profound lymphatic dysfunction [112], consistent with venous leg ulcers as the most common non-cancer-linked comorbidity for LE [113]. Indeed, chronic venous insufficiency is the most common cause of lower extremity LE, accounting for approximately 40% of lower extremity LE cases [114]. The etiology of lymphatic failure in venous disease is not well understood, but recent NIRF-LI evidence depicts dermal backflow uniquely in ankle regions associated with ulcer formation, even before ulcer formation occurs [112]. In addition, disease stage appears to correlate with the degradation of lymphatic function, with increased dermal backflow and impaired contractile function occurring with progression of venous disease [115].

### 4.2. Inflammatory/Rheumatological Diseases

**Figure 11:**
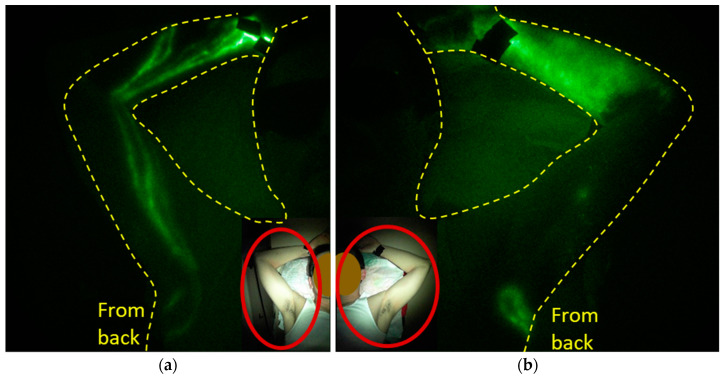
(**a**) Unaffected and (**b**) affected arms of a subject with chronic joint pain with suspected autoimmune disorder. Injection sites are covered with round bandages and/or black vinyl tape. The brightness and contrast of the images have been adjusted to facilitate the visualization of the 16-bit imaging depth.

Lymphatic dysfunction has also been linked to rheumatoid arthritis [116,117,118,119], with LE a known comorbidity [120] and dermal backflow evident on lymphoscintigraphy [121]. Other inflammatory disorders are also associated with LE [122,123], suggesting that, again, the pro-inflammatory state is associated with lymphatic dysfunction. A mechanism that may be responsible for impairment of lymph drainage in autoimmune disorders could be neutrophil recruitment to subcapsules of draining LNs in inflamed peripheral tissues [124]. Rheumatoid arthritis patients have elevated numbers of neutrophils that participate in the process of NETosis (neutrophil extracellular traps, NETs) induced by proinflammatory cytokines, including TNF-α. NETosis occurs when neutrophils spew DNA nets that display autoantigens [125] and can intravascularly trap cells and cellular debris to play a critical role in hemovascular thrombi formation [126]. Whether NETosis plays a similar role in the lymphatic vasculature during inflammatory conditions remains to be seen. However, Figure 11 is an example of NIRF-LI images of dermal backflow and distal flow in the affected hand of a subject never treated for cancer but possessing chronic joint pain with suspected autoimmune disorder. This suggests that the early phenotypes of sub-clinical LE may not be unique to cancer-acquired LE and that cancer-acquired LE may share aspects of etiology with autoimmune conditions.

### 4.3. Cellulitis

Cellulitis, a complication of LE, is an inflammatory event in the inner layers of skin, occurs in approximately 25–50% of LE patients, and in 2.5% of the general population [127,128,129,130]. This complication of LE is expensive--more than $3.7 billion was spent on 240,000 inpatient admissions for cellulitis in the general United States population in 2004 [131], and more than 14.5 million outpatient visits for skin infections, including cellulitis, occurred in 2005 [132]. Cellulitis accounts for 10% of all infectious disease-related hospital admissions in the US [133] and presents as a painful area of redness, without pus, accompanied by fever and fatigue. Cellulitis can spread hematogenously to other areas of the body (a process known as “blood poisoning”), can progress to sepsis, and has long been believed to result when normal skin flora enter the dermal layers due to a break in skin [134,135]. *Staphylococcus aureus* and *Streptococcus* groups A, G, and B have been blamed as the causative bacteria in most cellulitis cases [136,137].

The established dogma for cellulitis states that LE patients experience cellulitis because of diminished infection clearance. Pathogenic infection is accompanied by the release of pro-inflammatory cytokines that allow further damage to lymphatic vessels and increased limb swelling, thus creating a vicious cycle. Protein-rich lymphatic fluid, which is a feature of LE, is believed to provide a fertile medium for bacterial growth [138]. These established notions of cellulitis etiology appear to relate well to what is known about LE. Curiously though, bacterial cultures from cellulitis exudates rarely grow [139], and skin breaks are not always observed at cellulitis onset. The discrepancy between the low prevalence rate of microorganisms and the severity of clinical symptoms suggests that the mechanism of lymphedema-related cellulitis is not one of simple infection.

Of note, 30% of cellulitis is misdiagnosed [133], and NIRF-LI could help differentiate cellulitis (affecting dermis), abscesses (which affect the epidermis), and deeper wounds, as lymphatic vessels surrounding wound sites may exhibit distinct patterns and drainage, depending on wound type. This information could provide direction for optimal treatment (specific antibiotics, compression versus no compression, duration of treatment).

Stagnation in lymph vessels most likely contributes significantly to cellulitis, because moving lymph with massage-mimicking advanced pneumatic compression devices in LE patients lowered rates of cellulitis episodes from 21.1% to 4.5% in one study [140] and from 0.26 episodes to 0.05 episodes per year in another study [141]. The eradication of cellulitis after LVB/VLNT surgeries, however, awaits a mechanistic explanation.

## 5. Conclusions and Perspectives

The lymphatic vasculature is an open, unidirectional system providing afferent and efferent paths for mounting and resolving immune responses and clearing inflammatory waste products. Cancer progression and treatment directly involve the lymphatics and therefore can be expected to acutely impact immune responses as well as lymphatic function. Using a technique which we developed nearly two decades ago, we show that the earliest phenotype of lymphatic dysfunction occurs in breast and head and neck cancer patients before and during cancer treatment, and in some cases, can be resolved with complementary medicine approaches. Still, in other cases without intervention, early dysfunction progresses and persists for months to years even without the accepted clinical symptoms of LE. Because changes in lymphatic function seen before and after cancer treatment are also observed with other chronic conditions, one might speculate that etiological aspects of LE may be shared in common with some of these chronic conditions. It remains to be demonstrated whether we can “cure” LE by interceding early, before the onset of irreversible clinical symptoms, as we attempt to intercede before venous ulcer formation in patients with venous disease or before irreversible bone loss in patients with rheumatoid arthritis.

The lymphatic vasculature is an essential part of the immune system that is involved in chronic conditions (including cancer) that are associated with the process of aging. The lack of routine approaches to image the lymphatics has limited our understanding of its role in diseases, as revealed in NIRF-LI studies of lymphatic dysfunction of cancer progression and treatment. In the hemovascular system, vascular interventions were not enabled until the advent of angiography. In the case of the lymphatics, the predominantly clear lymph and transparent lymphatic vessels escape notice in clinical surgery practice or in preclinical studies, further contributing to the lack of understanding of the role and responses of the lymphatics to disease conditions and treatment. It is highly likely that the changes in lymphatics with disease progression and treatment seen in cancer-acquired lymphedema may be shared by other chronic diseases that involve the immune system. The ability to clinically evaluate lymphatic function in “point-of-care” studies could accelerate more effective strategies to treat these diseases. For example, we recently used NIRF-LI techniques to demonstrate the lymphatic delivery of a TNF inhibitor to restore contractile lymphatic function in an animal model of rheumatoid arthritis [118] and of a checkpoint blockade inhibitor to more effectively suppress primary tumor growth and arrest metastasis than from systemic administration [142]. The ability to develop more effective treatments for chronic conditions requires the ability to visualize the function and anatomy of the lymphatics in disease progression and in response to treatment.

## Supplementary Materials

The following are available online at https://www.mdpi.com/2072-6694/12/8/2280/s1, Video S1: Lymphatic flow in the arm of a healthy control subject, Video S2: Lymphatic flow in the feet of a subject with bilateral lymphedema.
